# Substitutes of structural and non-structural autologous bone grafts in hindfoot arthrodeses and osteotomies: a systematic review

**DOI:** 10.1186/1471-2474-14-59

**Published:** 2013-02-07

**Authors:** Marc Andreas Müller, Alexander Frank, Matthias Briel, Victor Valderrabano, Patrick Vavken, Vahid Entezari, Arne Mehrkens

**Affiliations:** 1 Orthopedic Department University Hospital Basel, Spitalstrasse 21, Basel, 4031, Switzerland; 2 Basel Institute for Clinical Epidemiology and Biostatistics, University Hospital Basel, Hebelstrasse 10, Basel, 4031, Switzerland; 3 Department of Clinical Epidemiology and Biostatistics, McMaster University, 1280 Main Street West, Hamilton, ON, L8S 4K1, Canada; 4 Center for Advanced Orthopedic Studies, Beth Israel Deaconess Medical Center and Harvard Medical School, Brookline Avenue 330, Boston, MA, 02215, USA

## Abstract

**Background:**

Structural and non-structural substitutes of autologous bone grafts are frequently used in hindfoot arthrodeses and osteotomies. However, their efficacy is unclear.

The primary goal of this systematic review was to compare autologous bone grafts with structural and non-structural substitutes regarding the odds of union in hindfoot arthrodeses and osteotomies.

**Methods:**

The Medline and EMBASE and Cochrane databases were searched for relevant randomized and non-randomized prospective studies as well as retrospective comparative chart reviews.

**Results:**

10 studies which comprised 928 hindfoot arthrodeses and osteotomies met the inclusion criteria for this systematic review. The quality of the retrieved studies was low due to small samples sizes and confounding variables. The pooled random effect odds for union were 12.8 (95% CI 12.7 to 12.9) for structural allografts, 5.7 (95% CI 5.5 to 6.0) for cortical autologous grafts, 7.3 (95% CI 6.0 to 8.6) for cancellous allografts and 6.0 (95% CI 5.7 to 6.4) for cancellous autologous grafts. In individual studies, the odds of union in hindfoot arthrodeses achieved with cancellous autologous grafts was similar to those achieved with demineralised bone matrix or platelet derived growth factor augmented ceramic granules.

**Conclusion:**

Our results suggest an equivalent incorporation of structural allografts as compared to autologous grafts in hindfoot arthrodeses and osteotomies. There is a need for prospective randomized trials to further clarify the role of substitutes of autologous bone grafts in hindfoot surgery.

## Background

Hindfoot arthrodeses and osteotomies are performed to treat various pathologies such as posterior tibial tendon insufficiency [1-3], hindfoot osteoarthritis [4-6] and deformities related to posttraumatic conditions [7,8] and neuromuscular disease [9,10]. In these procedures, structural (cortical) autologous bone grafts and substitutes are used when an open wedge osteotomy or an interposition arthrodesis is performed to provide mechanical support in the resulting bone gap. On the other hand, non-structural (cancellous) bone grafts and substitutes are utilized for in situ arthrodesis with the intention to promote bone healing.

The hindfoot is a challenging environment for bone healing due to mechanical loading, restricted blood supply and thin soft tissue coverage. Non-unions are observed in up to 14% [11-14] of patients undergoing hindfoot fusions and osteotomies and even more frequently in patients with impaired bone healing, such as diabetics and smokers [4,15,16]. Implanted structural bone grafts may also show collapse under load with consecutive loss of deformity correction [17]. Both, non-union and graft collapse may lead to unsatisfactory outcomes [12,18]. Therefore, the selection of an appropriate bone graft able to withstand these challenges is important in hindfoot arthrodeses and osteotomies.

From a biological standpoint autologous grafts are an excellent choice. They incorporate quickly due to favourable osteoconductivity [19-21] and osteoinductivity [22] as well as potential osteogenicity [20,23]. Moreover, they display remarkable biomechanical resistance [24]. However, the use of autologous grafts is limited by associated donor site morbidity [25], increased surgery time and finite availability [26-28]. These limitations incline surgeons to use substitutes for autologous bone grafts. Allografts are commonly the first option with similar osteoconductivity and osteoinductivity as compared to autologous grafts [29], but they carry the risk of transmitting infectious diseases [30] and may incite an immunological response [31,32] leading to impaired graft incorporation [33,34] and secondary graft collapse [35]. Further processing of allografts using freeze drying techniques and treating the graft with hypotonic solutions, acetone, ethylenoxide or gamma irradiation can eliminate cellular and viral particles and thus can lower the risk of infectious disease transmission [36-38]. These acellular allografts preserve their osteoconductive properties but studies showed that their mechanical strength and osteointegration may be affected by this process [39].

Alternatively, allografts can be decalcified to extract demineralized bone matrix (DBM) which is known to have osteoinductive and partly osteoconductive properties [40]. However, the osteoinductivity of DBM depends on multiple factors including the manufacturing process [41,42] and the donor [43]. More consistent characteristics may be expected from synthetic substitutes of autologous grafts. Current market options range from very brittle ceramics (made from hydroxapatite [HA] or tricacliumphosphate [TCP]) that have favourable osteoconductive properties [44] to more resistant osteoconductive metals such as tantalum [45] as well as costly recombinants of bone morphogenetic proteins (BMPs) [46] and platelet derived growth factors (PDGFs) [47].

Nonetheless, the choice between autologous grafts and different substitutes should not only be based on biologic and biomechanical considerations, but also on the best evidence available in the literature. Thus, the primary goal of this systematic review was to assess the efficacy of autologous bone grafts and their structural and non-structural substitutes to achieve union in hindfoot arthrodeses and osteotomies. As a secondary goal, we aimed to compare the resistance of structural grafts to collapse in these hindfoot procedures.

## Methods

### Search strategy

We conducted an electronic search of Medline, the Cochrane Library, and EMBASE from inception of each database to December 2011 for randomized and non-randomized, controlled studies on the use of structural and non-structural autologous bone grafts and substitutes in hindfoot osteotomies and arthrodeses. We used “bone transplantation”, “allograft”, “xenograft”, “synthetic bone graft”, “beta tricalcium phosphate”,”hydroxyapatite”, “bone morphogenetic protein”, “demineralized bone matrix”, “foot” as terms or medical subject headings. There were no restrictions with respect to language or date of publication.

Studies eligible for this systematic review had to compare cortical or cancellous autologous bone grafts with any structural or non-structural substitute in patients undergoing subtalar, talonavicular, triple arthrodesis or lateral column lengthening. Only prospective or retrospective, controlled trials with a minimum sample size of 20 patients were eligible for inclusion. All titles and abstracts identified by our search were reviewed by two orthopedic surgeons independently (AF, AMM). If either reviewer deemed an abstract potentially eligible, the full text of that article was obtained and assessed for eligibility by both reviewers. Disagreement was resolved by discussion or consultation with the senior author.

Two reviewers independently extracted patient demographics, operative data, and outcome data from each article. The primary outcome data comprised of rate of union. Secondary outcome data referred to the occurrence of graft collapse either defined as an obvious disintegration of the graft or measured indirectly by a loss of hind- and/or midfoot alignment. In addition, time-to-union was recorded.

### Quality assessment of retrieved studies

The methodological quality of included studies was independently assessed by two reviewers (AMM, AM). Randomized controlled trials were assessed based on concealment of treatment allocation, blinding of outcome assessors, completeness of follow-up, and the attainment of sufficient power to detect significant differences. The Newcastle-Ottawa Quality assessment scale [48] was used for the quality assessment of non-randomized studies. In this context, the included studies were evaluated according to three main categories, i.e. “selection”, “comparability” and “outcome”. Each of these categories comprised several evaluation criteria: Within the category “selection”, each study was assessed regarding the representativeness of the (1) exposed and the (2) unexposed study cohort and (3) the ascertainment of exposure to the surgical procedure and graft of interest. It was also verified whether (4) the outcome of interest was absent at the beginning of the study. Within the category “comparability”, it was assessed if the study controlled for confounding variables. Within the category “outcome”, each study was tested for (1) blinded or record linked outcome assessment and (2) completeness of follow-up (i.e. a documented follow-rate ≥ 80%). It was also assessed if (3) the follow-up time was long enough to detect the outcome of interest.

As a final summary, stars were allotted to each of the three main categories with each star representing a fulfilled evaluation criterion. A maximum of two stars could be allotted to the category “comparability”.

### Statistical analyses

Data for the primary endpoint *rate of union* could be gathered from all but one included study. Using these data, the odds for union were calculated for structural and non-structural autologous grafts and allografts, independently. For data synthesis, odds were pooled using inverse variance weights in a fixed effects model, and assessed for mathematical between-study heterogeneity using Q statistic. The cut-off was set at a p-value of 0.1 to account for loss of power due to small sample sizes. In case of significant mathematical heterogeneity, the DerSimonian-Laird random effects model [49] was used. This method allows for between-study heterogeneity assuming that the individual study results are different, but normally distributed around a common, pooled effect. We used Egger’s regression [50] to test for publication bias.

Since data were collected by type of graft and not by study, the odds rather than the odds ratio are given. All results are given as odds with the 95% confidence interval (CI). All calculations were done with intercooled STATA 12 (StataCorp LP, College Station, Tx).

## Results

### Literature search

**Figure 1:**
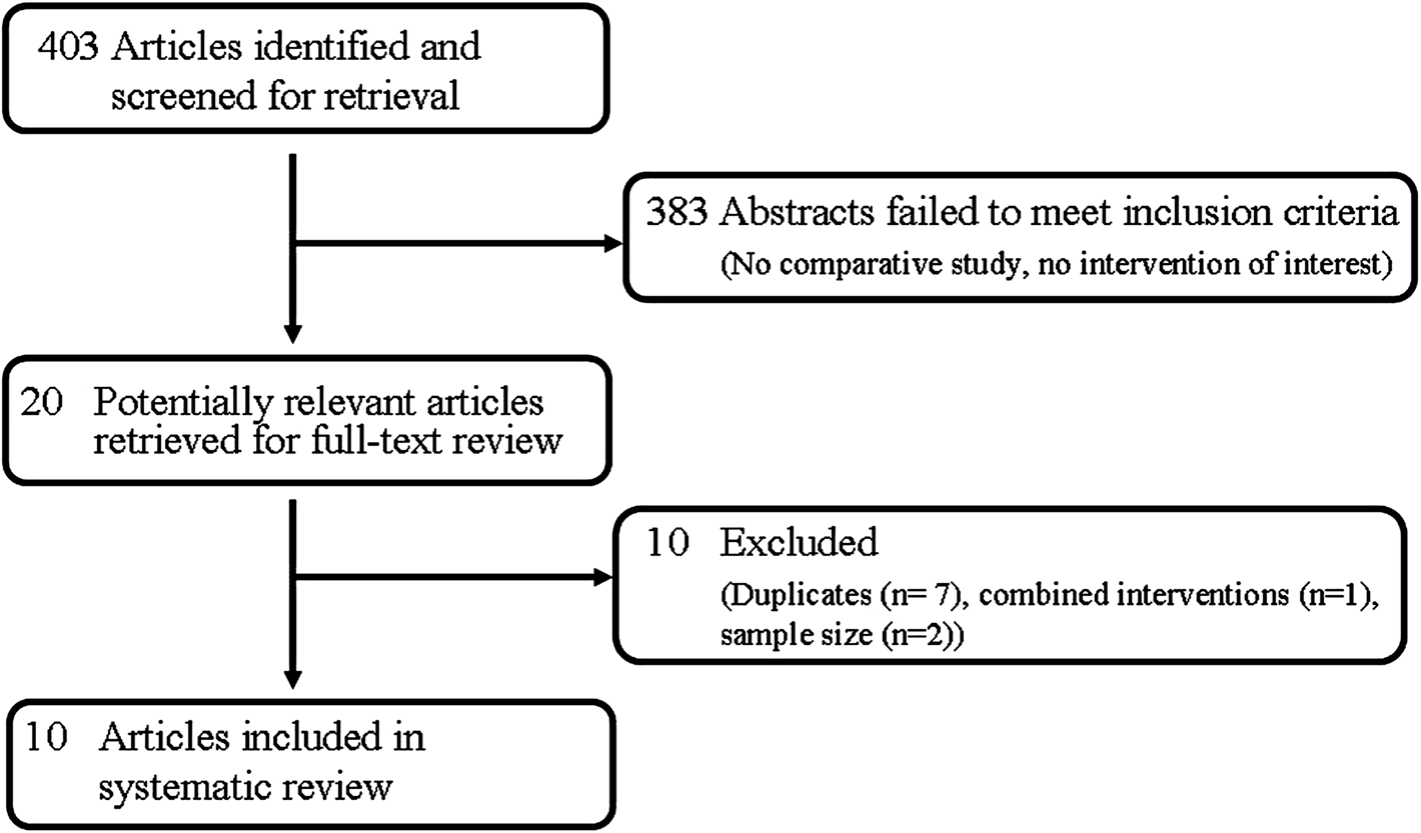
Study flow diagram.

Our online search produced 403 articles in total. After exclusion of duplicates and studies without a control group, 10 studies met our eligibility criteria (Figure 1). Among the ten included studies, seven compared structural autologous bone grafts with substitutes (Table 1). One randomized controlled trial [51] and two retrospective chart reviews [52,53] compared structural allografts with autologous grafts in Evan’s osteotomies of the calcaneus. Another three retrospective chart reviews [17,54,55] compared structural allografts with autologous grafts in mixed patient populations that comprised both hindfoot arthrodeses and osteotomies. A subgroup analysis in a retrospective chart review [4] compared structural allografts with autologous grafts in isolated hindfoot arthrodeses.

**Table 1:** Studies comparing structural autologous bone grafts with substitutes in hindfoot arthrodeses and osteotomies

**Ref.**	**Methodology**	**Results**	**Quality**
***Structural allografts *versus *cortical autologous grafts in hindfoot OTs***			
**Dolan et al**. [51]	**Randomized controlled trial**	**Rate of union at 8 weeks**	No concealed allocation No blinded outcome assessment 100% FU Underpowered study
	18 freeze-dried structural allografts vs. 15 cortical autologus grafts in 31 adults undergoing Evan’s OTs. FU: 8 and 12 weeks	Allografts: 17/18 (94%)	
		Autologous grafts: 9/15 (60%), P = 0.03	
		**Rate of union at 12 weeks**	
		100% for allografts and autologous grafts	
		**No graft collapse** in both groups	
**Templin et al**. [52]	**Retrospective comparative chart review** (1994–2003)	**Rate of union**	Selection: ***
		Allografts: 27/30 (90%)	Comparability:
	30 freeze-dried structural allografts vs 5 structural autologous grafts in 35 children undergoing Evan’s OTs. Mean FU 3.6 years (range 6–12 years)	Autologous grafts: 4/5 (80%)	Outcome: *
		P = n. s.	
**Kwak et al**. [53]	**Retrospective comparative chart review** (2000–2005)	**Talo-1**^**st **^**metatarsal, talo-calcaneal and calcaneal pitch angle at final FU**	Selection: ***
			Comparability:
	118 acellular allografts (Tutoplast ®) vs. 10 structural autologous grafts in 79 children undergoing Evan’s OTs. Mean FU 15 months (range 13-21months)	No significant difference between the two graft types	Outcome: *
***Structural allografts *versus *cortical autologous grafts in hindfoot ADs***			
**Easley et al**. [4]	**Subgroup comparison in a retrospective chart review** (1988–1995)	**Rate of union**	Selection: ***
		Allografts: 2/5 (40%)	Comparability:
	5 structural allografts vs. 29 structural autologous grafts in isolated subtalar ADs. Mean FU 51 months (range 24–130 months)	Autologous grafts: 24/29 (83%)	Outcome: *
		P = n.s.	
		**Time to union**	
		Autologous grafts: 16 weeks (10-30 weeks)	
		P = n. s.	
***Structural allografts *versus *cortical autologous grafts in miscellaneous procedures (hindfoot ADs/OTs)***			
**Grier et al.**[54]	**Retrospective comparative chart review** (1996–2006)	**Rate of union**	Selection: **
		Allografts + PRP: 29/31 (94%)	Comparability:
	31 structural freeze-dried allografts + PRP vs. 20 structural autologous grafts in 18 adult Evan’s OTs and 33 adult CC ADs. Mean FU: 20 months (range 3-72 months)	Autologous grafts: 14/20 (70%)	Outcome: *
		P = 0.045	
		**Improvement of the talo-1**^**st **^**metatarsal and calcaneal pitch angle**	
		No significant difference between the two graft types	
**Danko et al**. [17]	**Retrospective comparative chart review** (1990–1992)	**Graft collapse**	Selection: ***
		*Evan’s OT:*	Comparability:
	7 structural allografts vs 33 structural autologous grafts in 69 pediatric Evan’s OTs and 61 pediatric CC ADs. Mean FU 2.5 years (range 0.6-7.8 years)	Allografts: 0/39	Outcome: ***
		Autologous grafts 0/30	
		P = ?	
		*CC ADs*:	
		Allografts: 17/58 (29%)	
		Autologous grafts: 0/3 (0%)	
		P = ?	
**Mahan et al.**[55]	**Retrospective comparative chart review** (1977–1990)	**Rate of union**	Selection: **
		Allografts: 198/215	Comparability:
	215 freeze dried allografts vs 85 autologous grafts in 153 OTs, 55 ADs, 82 other procedures. Minimum FU 6 weeks	(92%)	Outcome: *
		Autologous grafts: 78 /85 (92%),	
		P = n. s.	
		**Rate of delayed union**	
		Allografts: 8/215 (4%)	
		Autologous grafts 2/85 (2%)	
		P = n. s.	

Abbreviations: CC=calcaneocuboideal, OTs= osteotomies, ADs= arthrodeses, FU = follow-up, n.s.= not significant.

Four studies on non-stru bone grafts met our inclusion criteria (Table 2) which all included subjects undergoing hindfoot fusions. There was one randomized controlled trial comparing recombinant PDGF augmented ceramic granules with cancellous autologous grafts [56]. In a prospective controlled study [57], DBM was compared with cancellous autologous grafts. The remaining two retrospective chart reviews [4,58] compared cancellous allografts and no graft application with cancellous autologous grafts.

**Table 2:** Studies comparing non-structural autologous bone grafts with substitutes in hindfoot arthrodeses and osteotomies

**Ref.**	**Methodology**	**Results**	**Quality**
***Cancellous allografts *versus *cancellous autologous grafts in hindfoot ADs***			
**McGarvey et al.**[58]	**Retrospective comparative chart review** (1990–1992)	**Rate of union**	Selection: ***
		Allografts: 21/24 (88%)	Comparability:
	24 acellular allograft chips vs 17 cancellous autologous grafts in 37 subtalar, double and triple ADs. FU: Minimum 18 months	Autologous grafts: 16/17 (94%)	Outcome:*
		P=?	
		**Time-to- union**	
		*Triple/Double/Subtalar AD*	
		Allografts: 4.0/4.0/4.1 months	
		Autologous grafts 3.0/3.2/3.6 months	
		P= n. s	
**Easley et al.**[4]	**Subgroup comparison in a retrospective chart review** (1988–1995)	**Rate of union**	Selection: **
		Allografts: 14/17 (82%)	Comparability:
	17 cancellous allograft vs. 94 cancellous autologous grafts in subtalar ADs. Mean FU 51 months(range: 24–130 months)	Autologous grafts: 80/94 (85%)	Outcome:*
		P= n. s.	
		**Time –to- union**	
		Allografts: 13 weeks	
		(10–24 weeks)	
		Autologous grafts: 11weeks (8–20 weeks)	
		P= n. s	
***DBM *versus *cancellous autologous grafts in hindfoot ADs***			
**Michelson et al.**[57]	**Prospective comparative study** (1990–1993)	Rate of union	Selection: ***
	37 DBM vs.18 cancellous autologous grafts in 11 subtalar AD’s, and 44 triple AD’s FU: Until complete healing	DBM: 36/37 (97%)	Comparability:
		Autologous grafts: 16/18 (89%)	Outcome:***
		P= n. s.	
		Time –to-union	
		DBM: 3.0 – 3.4 months	
		Autologous grafts 2.7-3.7 months	
		P=n. s.	
***No graft *versus *cancellous autologous grafts in hindfoot ADs***			
**Easley et al**. [4]	**Subgroup comparison in a retrospective chart review** (1988–1995)	**Rate of union**	Selection: **
		No graft: 34/39 (87%)	Comparability:
	39 “no graft” vs 94 cancellous autologous grafts in isolated subtalar ADs. Mean FU 54 months (range: 24–130 months)	Autologous grafts: 80/94 (85%)	Outcome:*
		P= n. s.	
		**Time-to-union**	
		No graft: 11w (8–24)	
		Autologous grafts: 11weeks (8–20 weeks)	
		P= n. s	
***Synthetic bone grafts *versus *cancellous autologous grafts in hindfoot ADs***			
**DiGiovanni et al**. [56]	**Randomized controlled trial** (2006/7)	**Rate of X ray based union at 6-12-36 weeks**:	No concealed allocation Blinded outcome assessment 80-93% follow-up Underpowered study
	14 PDGF augmented ß-TCP (Augment ®) vs 6 cancellous autologous grafts in 20 adult subtalar, triple, ankle ADs. FU:6, 12 and 36 weeks		
		PDGF/ß-TCP:	
		0/11(0%)-5/12(42%)-10/13	
		Autologous grafts:	
		0/4 (0%)-1/3 (33%)-3/5 (60%)	
		P= ?	
		**Rate of CT based union at 6–12 weeks**	
		PDGF/ß-TCP:	
		5/13 (38%)-9/13% (69%)	
		Autologous grafts:	
		2/5 (40%)- 3/5 (60%)	
		P= ?	

Abbreviations: OTs= osteotomies, ADs= arthrodeses, FU= follow-up, n.s.= not significant.

In nine [4,51-58] of the ten included studies rate of union was used as an outcome measure to compare autologous grafts with structural or non-structural substitutes. In all of these nine studies union was defined on the basis of plain radiography or conventional tomography as a bridging trabeculation across the osteotomy gap or arthrodeses site. Two studies [51,57] used absence of pain at the surgical site as an additional criterion for union. In only one study [56] healing was additionally assessed using computed tomography (CT) with union defined as a continuity of the trabecular lines within 50% of the (joint) space to be bridged.

In four [17,51,53,54] of the ten included studies graft collapse was used as primary or secondary outcome measure. All of these studies compared structural allografts with autologous grafts in pediatric [17,53] or adult [51,54] lateral column lengthening procedures. Graft collapse was defined on plain radiographs either directly as obvious graft disintegration [17,51] or indirectly based on the associated change of the talo-first metatarsal, talo-calcaneal and calcaneal pitch angle [53,54].

The quality of retrieved studies is summarized in Table 1 and 2. In brief, all studies had small sample sizes at least in one group and thus limited capacity to detect significant differences. In both randomized controlled trials, patient allocation was not concealed and only in the trial by DiGiovanni et al. [56] outcomes were assessed by blinded observers. All non-randomized trial did not control for confounding factors.

### Rate of union: structural autologous grafts and allografts

Data could be abstracted for 154 implanted structural autologous grafts. The pooled, fixed effects odds of union across all studies for structural autologous grafts was 6.5 (95% CI 6.0 to 7.0), but showed significant heterogeneity (p<0.001). Using the DerSimonian-Laird random effects, we calculated the pooled odds of union for autologous grafts to be 5.7 (95% CI 5.5 to 6.0), which is significantly different from no effect (or odds of 1) at a p-value of less than 0.001. There was no evidence for publication bias (p=0.678).

For structural allografts, we could collect data on 299 cases. The pooled, fixed effects odds for union were 14.5 (95% CI 14.0 to 14.0), but again with significant heterogeneity (p<0.001). The random effects odds were 12.8 (95% CI 12.7 to 12.9), which is significantly different from no effect (or odds of 1) at a p-value of less than 0.001. There was some evidence for publication bias (p=0.037), suggesting overestimated odds.

The direct comparison of structural autologous grafts and allografts show that the 95% confidence intervals for union do not overlap, which is indicative of a significant difference.

### Rate of union: non-structural autologous grafts and allografts

In order to quantitatively assess the rate of union for non-structural autologous grafts, data were abstracted from 228 hindfoot fusions. The pooled fixed odds for union were 6.0 (95% CI 5.7 to 6.4). Again, there was significant heterogeneity (p<0.001). The random effects odds were 7.3 (95% CI 6.0 to 8.6), again significantly different from no effect (or odds of 1) with a p-value of less than 0.001. There was no evidence for publication bias (p=0.291).

For non-structural allografts, data from only 41 hindfoot fusions from two studies [4,58] could be included into analysis. The pooled fixed odds for union were 5.9 (95% CI 5.0 to 6.7) with again significant heterogeneity (p=0.016). The random effect odds for union with non-structural allografts were 5.8 (95% CI 4.4-7.3).

### Rate of union: other non-structural grafts

For the remaining non-structural grafts data could only be obtained a single study. For these graft types odds for union were calculated based on the individual study results. The odds of union were 6.8 (95% CI 5.9 to 7.7) for patients receiving no graft, 8.0 (95% CI 6.5 to 9.5) for patients receiving DBM and 3.7 (95% CI 2.4 to 5.0) for patients receiving PDGF/ß- TCP.

### Graft collapse

In all four studies with graft collapse as an outcome measure the use of structural allografts was not associated with a significantly increased graft disintegration or loss of midfoot alignment as compared to structural autologous grafts.

## Discussion

The primary goal of this systematic review was to review the evidence for the efficacy of structural and non-structural autologous bone grafts and substitutes with respect to rate of union in hindfoot osteotomies and arthrodeses.

We acknowledge several limitations of this study. We pooled data from two different procedures- hindfoot osteotomies and arthrodeses- which may raise concerns from a methodological point of view. However, from a biological standpoint, arthrodeses and osteotomies are very similar situations since in both procedures the bone graft is placed in between fresh bleeding osseous surfaces. We also found major methodological limitations in all studies included in this review. Particularly, small sample sizes and confounding variables were major points of criticism which makes the direct comparison of different grafts types difficult. Therefore, we decided not to report odds ratios, but odds for the rate of union achieved with the individual graft types. In addition, all studies except two [56,58] assessed the presence of bony healing on the basis of plain radiographs which are limited in studying the bony consolidation within the complex anatomy of a fused hindfoot joint or a three dimensional osteotomy [12,59]. The rates of union which were assessed with the use of plain radiography might have been overestimated [12].

The quantitative data analysis performed in this systematic review suggests that structural allografts may be effective substitutes for autologous grafts in these procedures. The pooled random effect odds for union with structural allografts were 12.8 (95% CI 12.7 to 12.9) and thus even higher than those calculated for structural autologous grafts (i.e. 5.7 [95% CI 5.5 to 6.0]). Nevertheless, the limited quality of the underlying evidence as well as the presence of publication bias may have both inflated the odds for union in the allograft group. However, it is unlikely that more extensive and reliable evidence would change the odds of union for structural allografts to a value which is substantially below that of autologous grafts. Therefore, our findings at least suggest an equivalent incorporation of structural allografts as compared to autologous grafts. This conclusion from our quantitative data analysis may further be supported by the fact that the pooled odds for union for cancellous allografts were also very similar to those calculated for cancellous autologous grafts.

One randomized controlled trial [17] and three retrospective chart reviews [17,52,53] also showed no increased risk for graft collapse when allografts were used instead of autologous grafts in Evan’s osteotomies, but these data were mainly generated in pediatric studies and thus definitively require further verification in a broad adult patient population.

Furthermore, the individual data from one prospective study [51] and from one small randomized controlled trial [57] indicated that DBM and PDGF augmented ß-TCP ceramic granules could potentially substitute for cancellous autologous grafts in hindfoot fusions. Nonetheless, with the lack of prospective studies with sound methodology, it currently remains unclear if the use of cancellous autologous bone grafts and substitutes is effective in promoting bony healing in arthrodeses of the hindfoot.

To our knowledge, this is the first systematic review on structural and non-structural bone grafts in hindfoot surgery, although many narrative reviews have already been published on this topic [46,60-63]. These narrative reviews mainly focused on the biologic properties of these grafts and did not synthesize results from different studies. On the other hand, there are several systematic reviews and meta-analyses on structural and non-structural autologous bone grafts and substitutes in spine surgery [64-70]. These studies showed conflicting results on the osteointegration of structural allografts. In one meta-analysis [70], structural allografts were associated with a higher frequency of graft collapse and non-union as compared to autologous grafts, but more recent meta-analysis [66] does not confirm these results.

## Conclusion

The current evidence suggests that structural allografts appear to be at least non-inferior to autologous grafts in respect to the odds for union in hindfoot arthrodeses and osteotomies. Considering the large number of bone grafts and substitutes used in hindfoot osteotomies and arthrodeses [71], there is an urgent need for well-designed randomized controlled trials to assess the efficacy of structural and non-structural autologous bone grafts substitutes.

## Abbreviations

DBM: Demineralised bone matrix; CI: Confidence interval; PDGF: Platelet derived growth factors; TCP: Tricalcium Phosphate.

## Competing interest

The authors declare that they have no competing interests.

## Authors’ contributions

The following authors have: designed the study: MAM, MB, AM, VV, gathered the data: MAM, AF, AM, analyzed and interpreted the data: PV, MB, VV; VE, written the initial draft: MAM, AM, VE, have given final approval of the version published: MAM, VV, AM, PV, AF. All authors read and approved the final manuscript.

## Pre-publication history

The pre-publication history for this paper can be accessed here:

http://www.biomedcentral.com/1471-2474/14/59/prepub
